# Immunometabolism of Phagocytes and Relationships to Cardiac Repair

**DOI:** 10.3389/fcvm.2019.00042

**Published:** 2019-04-11

**Authors:** Shuang Zhang, Gael Bories, Connor Lantz, Russel Emmons, Amanda Becker, Esther Liu, Michael M. Abecassis, Laurent Yvan-Charvet, Edward B. Thorp

**Affiliations:** ^1^Departments of Pathology and Pediatrics, Feinberg Cardiovascular and Renal Research Institute, Feinberg School of Medicine, Northwestern University, Chicago, IL, United States; ^2^UMR INSERM U1065/UNS, C3M, Bâtiment Universitaire ARCHIMED, Nice, France; ^3^Department of Pediatrics, Ann & Robert H. Lurie Children's Hospital of Chicago, Northwestern University, Chicago, IL, United States; ^4^Comprehensive Transplant Center, Northwestern Feinberg School of Medicine, Chicago, IL, United States

**Keywords:** macrophage, neutrophil, phagocyte, immunometabolism, hypoxia, reperfusion, cardiac repair

## Abstract

Cardiovascular disease remains the leading cause of death worldwide. Myocardial ischemia is a major contributor to cardiovascular morbidity and mortality. In the case of acute myocardial infarction, subsequent cardiac repair relies upon the acute, and coordinated response to injury by innate myeloid phagocytes. This includes neutrophils, monocytes, macrophage subsets, and immature dendritic cells. Phagocytes function to remove necrotic cardiomyocytes, apoptotic inflammatory cells, and to remodel extracellular matrix. These innate immune cells also secrete cytokines and growth factors that promote tissue replacement through fibrosis and angiogenesis. Within the injured myocardium, macrophages polarize from pro-inflammatory to inflammation-resolving phenotypes. At the core of this functional plasticity is cellular metabolism, which has gained an appreciation for its integration with phagocyte function and remodeling of the transcriptional and epigenetic landscape. Immunometabolic rewiring is particularly relevant after ischemia and clinical reperfusion given the rapidly changing oxygen and metabolic milieu. Hypoxia reduces mitochondrial oxidative phosphorylation and leads to increased reliance on glycolysis, which can support biosynthesis of pro-inflammatory cytokines. Reoxygenation is permissive for shifts back to mitochondrial metabolism and fatty acid oxidation and this is ultimately linked to pro-reparative macrophage polarization. Improved understanding of mechanisms that regulate metabolic adaptations holds the potential to identify new metabolite targets and strategies to reduce cardiac damage through nutrient signaling.

## Introduction

*According to the American Heart Association*, ~720,000 individuals in the United States alone will succumb to a first hospitalized acute myocardial infarction (AMI), and ~335,000 more will have a recurrent event (1). Improvements in timeliness and efficiency of clinical treatment have reduced mortality after first heart attack. Nevertheless, the incidence of heart failure, including post-MI heart failure, has seen recent escalations (2). The progression to heart failure is often related to the degree of ventricular damage after acute insult. Occlusion of coronary arteries restricts blood flow to the myocardium. This leads to reduced oxygen availability and metabolic substrates that are essential to sustain active cardiomyocyte metabolism. Oxygen restriction also modulates the immune response to the injured heart. Further clinical percutaneous reperfusion and re-oxygenation may cause unintended tissue damage in the ischemic heart (3).

*Acute Coronary Syndromes Have Long Been Associated With Inflammation* *(*4, 5) *and Metabolic Syndromes*. In the majority of patients who succumb to sudden death after AMI, cardiac pathology typically reveals significant accumulation of polymorphonuclear and mononuclear phagocytes. In general, acute inflammation is followed by a resolution program that acts to dampen the amplitude of inflammation and orchestrate tissue repair. However, in many diseases of aging, this transition fails and leads to a persistence of chronic inflammation (6). Defects in inflammation resolution are often linked to a systemic metabolic imbalance (7). A similar phenomenon may occur after AMI, in which inefficient inflammation resolution due to hyperlipidemia (8) is linked to maladaptive myeloid response (9). In experimental animals, the ischemic heart recruits a diverse repertoire of innate and adaptive immune cells. This is triggered by chemokines and cytokines that are secreted by cardiac resident and recruited cells. Neutrophils enter and accumulate in the ischemic heart soon after AMI, and this is followed by recruitment of monocyte subsets. Two monocyte sources are of particular interest in this context. This includes monocytes generated from hematopoietic stem cells in the bone marrow, and mature monocytes released from the splenic reservoir (10). Independent of their site of origin, these monocytes differentiate into cardiac macrophages (11), which may either contribute to promote or resolve the inflammatory response (11). This occurs by the liberation of cytokines and growth factors that are recognized by parenchymal and stromal cells and therefore modulate the tissue response to injury (12).

**Figure 1:**
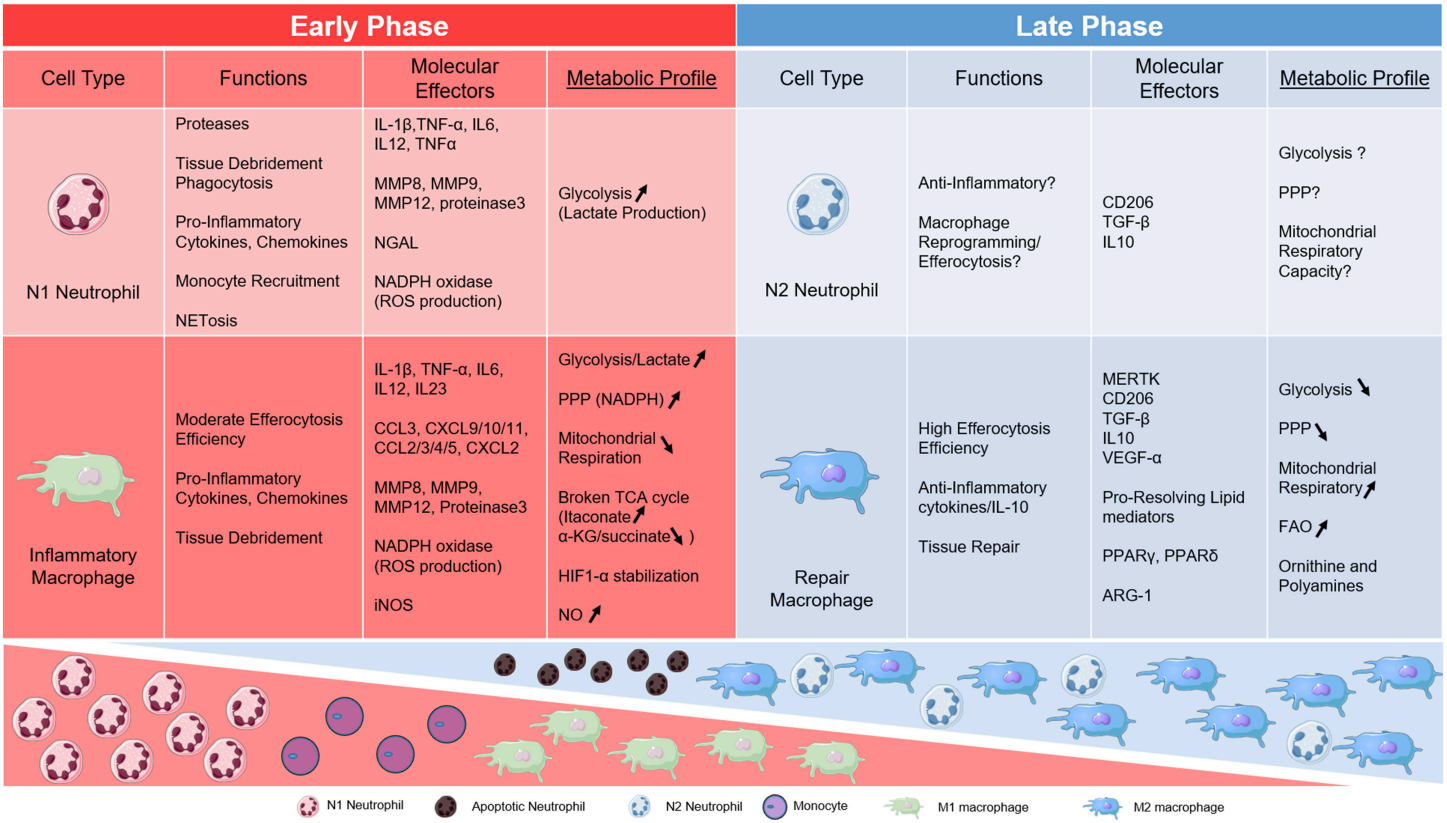
Table of metabolic links to key phagocytes during AMI. Neutrophil and Macrophage subsets are divided into early and late phases of cardiac inflammation after myocardial infarction and according to metabolic phenotype.

**Figure 2:**
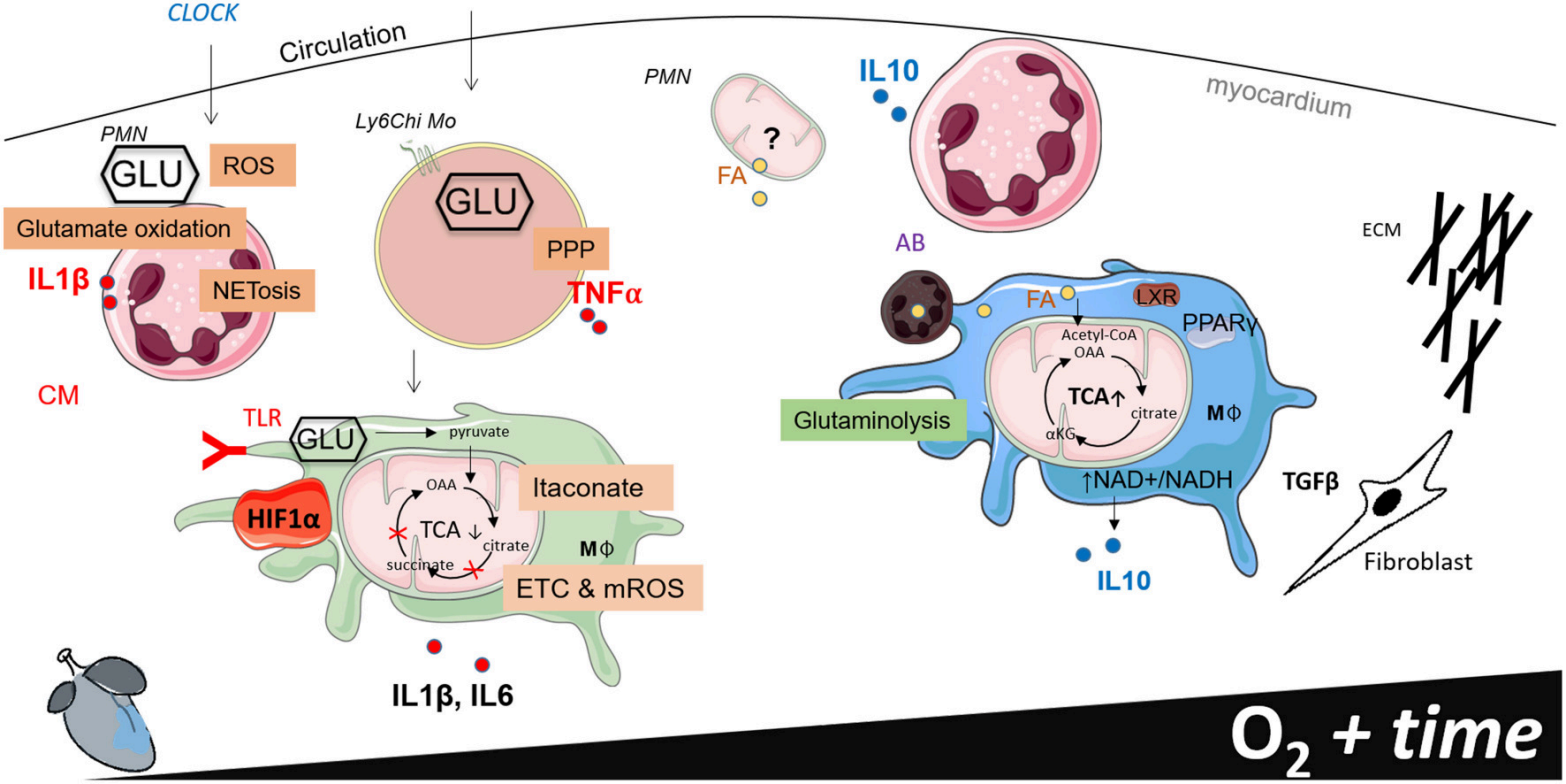
Working model of phagocyte immunometabolism after myocardial infarction (MI). This figure separates cardiac inflammation based on time (the first week post MI) and oxygen saturation within the infarct border zone. Little information is known about the functional metabolic capacity of macrophage CCR2 and MHCII resident and recruited subsets in the heart, therefore generalizations are made to classify macrophages according to metabolic phenotype.

*Within the ischemic myocardium*, reductions in both oxygen and nutrient supply are associated with acute cell death and necrosis (13). Prolonged ischemia impairs mitochondrial oxidative phosphorylation that is coupled to the synthesis of adenosine triphosphate (ATP) synthesis. In order to meet bioenergetic demands needs, cells must then rely on glycolysis, which in turn increases the concentration of lactate. Intracellular lactate accumulation lowers cytosolic pH whereas extracellular lactate is sensed by immune cells and can lead to their activation (14). Accumulation of metabolically active immune cells also depletes local oxygen and nutrient substrate availability. A consequence of reduced oxygen availability is the induction of hypoxia-inducible transcription factors (HIF), which act transcriptionally to promote glycolysis. Additional triggers, including liberation of damage associated molecular patterns (DAMP) in heart (15), activate immune cell toll like receptors, that are also tied to signaling that induce glycolytic polarization. This glycolytic switch has functional consequences. This includes facilitating the diversion of metabolites from Krebs cycle and the pentose phosphate pathway (PPP), to substrates that promote cellular proliferation during tissue repair. As the repair site matures, macrophages polarize to phenotypes associated with mitochondrial oxidative phosphorylation, as discussed below. Most studies of myocardial ischemic metabolism have focused on the myocyte. In contrast, discussed herein (Figures 1, 2) are advancements in the field of immunometabolism that hint at a significant role of phagocyte metabolism to cardiac repair.

## Neutrophil Immunometabolic Links to Heart

As the predominant phagocyte in blood, the glycolytic-biased polymorpho-nuclear (PMN) neutrophil, is primed to rapidly respond to secreted alarms of tissue injury (16). PMNs have been referred to as the “wrecking crew” for their propensity to secrete proteases that facilitate wound breakdown that enables wound clearance. Thus, PMNs contribute to reparative functions after tissue injury, however they are also notoriously affiliated with causing maladaptive collateral tissue damage, particularly after clinical reperfusion. Patients in the highest tertile of circulating neutrophils track with heightened risk for MI (17). This bad reputation extends to after cardiac transplant, as pre-operative neutrophil phenotype is a biomarker for early allograft rejection (18). Elevated neutrophils are also prognostic for poor clinical outcome (19) and directly correlate with infarct size following percutaneous coronary intervention (20). Neutrophil depletion and inhibition of neutrophil-derived enzymes (21) have long been associated with experimental improvements with cardiac function (22). Harmful neutrophil effects may be amplified in the setting of metabolic syndromes (23). For example, neutrophils from hyperlipidemic patients exhibit elevated markers of superoxide release (24). Moreover, neutrophils from diabetic humans and experimental mice are more susceptible to cell death by NETosis (neutrophil extracellular traps), which impairs wound healing (25) and is linked to glucose metabolism. In contrast, neutrophil depletion may aggravate cardiac function after AMI, which is consistent with a conserved reparative function (26). The mechanism underlying this protective role may be linked to crosstalk with macrophages, which can polarize toward a reparative phenotype after PMN efferocytosis. This dichotomy between deleterious vs. protective roles of PMNs after AMI may be better understood by examining PMN function at distinct stages of AMI inflammation vs. resolution vs. reperfusion, as further discussed below.

### Recent Reports Highlight our Evolving Understanding of Neutrophil Polarity (16)

For instance, neutrophils isolated from infarcted mouse hearts on the first days after coronary ligation expressed pro-inflammatory markers IL1β and TNFα. By contrast, the neutrophil profile was more anti-inflammatory a few days later (27). In tumors, neutrophils have been classified into N1 and N2 polarization states (28), akin to the widely but overly simplistic M1/M2 macrophage classification. In this context, it is tempting to speculate that neutrophil polarization states may reflect unique metabolic rewiring, as illustrated in the M1/M2 dichotomy.

Neutrophil variation also manifests through diurnal variation. For example, immune regulation may be tied to a metabolic clock (29). It has been estimated that ~15% of all metabolites may be under circadian control (30). This includes amino acid, lipid, and carbohydrate metabolic pathways (31). A molecular clock may integrate daily metabolic changes driven by feeding-fasting and energy storage, to immune function. Indeed, the master inflammatory regulator NFκB is required for maintenance of behavioral rhythm in mice (32). Among immune cells, neutrophils appear unique in their daily pattern of release, re-entry, and clearance from the bone marrow (33). In this context, neutrophil recruitment to sites of tissue injury is altered by time of day. In heart, elevations in chemokine-dependent recruitment of neutrophils occurred during non-sleep activity periods, relative to rest. During cardiac injury, heightened cardiac neutrophil infiltration was also observed during these activity periods (34). This was in parallel to systemic metabolic fluctuations in hepatic gluconeogenesis, elevated serum glucose (35), and leukocyte mobilization from the bone marrow (36). Nevertheless, it remains unclear if rhythmic neutrophil recruitment is also associated with neutrophil metabolic and inflammatory polarization. Although causal molecular clock mechanisms in neutrophils have not been characterized in detail, clock genes such as *Bmal1* exhibit significant daily variation in human neutrophils. Furthermore, human peripheral neutrophil pools display daily oscillation that correlates with time-dependent changes in superoxide production and phagocytosis (37). Circadian clock also controls the activity of the NAD+ dependent deacetylase *Sirtuin3*, which dampens mitochondrial respiration and oxidative enzyme activity (38). Interestingly, Sirtuin 3 deficiency aggravates experimental thrombosis and leads to increased neutrophil extracellular traps (NET). Taken together, intriguing links between circadian rhythm, neutrophil function, and metabolism are brought to light (39).

### Recent Findings Implicate Mitochondrial Respiration Pathways in Neutrophil Maturation

For example, autophagy-dependent generation of fatty acids is important during neutrophil differentiation. Autophagy-defective neutrophil precursors have increased glycolytic activity but impaired mitochondrial respiration and fatty acid oxidation. This led to lipid droplet accumulation and diminished neutrophil development (40). These data suggest that autophagy is necessary for the release of free fatty acids from intracellular stores and within neutrophil precursor cells. That is, lipophagy may be required to provide mitochondrial fuel to support oxidative phosphorylation necessary for neutrophil maturation. On the other hand, mice lacking autophagy related 5 (*Atg5)*, a gene essential for autophagosome formation, exhibited increased neutrophil proliferation and maturation (41). Furthermore, treatment of myeloid progenitors with ATP-synthase inhibitor oliogomycin, reduced electron transport chain flow and neutrophil differentiation, highlighting the essential role of oxidative phosphorylation during neutrophil maturation (42). Finally, mitochondrial dysfunction has been associated with an impaired unfolded protein stress response and mitochondrial dysfunction, leading to reduced neutrophil differentiation (43, 44). The contribution of most of these pathways in heart are largely untested.

### As Early as the 1960s, Observations Were Made in Guinea Pigs, Reporting That Mature Polymorphonuclear Leukocytes Are Glycolytic (45)

This is consistent with ultrastructural imaging, which often fails to identify significant numbers of mitochondria in mature neutrophils (46). Furthermore, mitochondrial respiration in neutrophils was found to be low (47). These observations have led to a hypothesis of high glycolytic reliance and a selective role of the mitochondria as a platform for apoptotic signaling (47, 48). Indeed, neutrophils rely greatly on glycolysis for their inflammatory functions (48). Glycolysis is increased in neutrophils during phagocytosis, and in the absence of glucose, the rate of ATP generation in neutrophils drops drastically. Furthermore, neutrophils from patients with chronic granulomatous disease have the same rate of glycolysis and ATP content as normal cells, consistent with resistance to defective respiration with glycolytic bias (49). After release, NETs become crosslinked to plasma fibrinogen, leading to thrombosis. This is a reason for the “no-reflow” phenomenon observed when artery circulation is restored. Thus, an experimental treatment approach employing DNase-based and thrombolytic agent combinations to treat ischemia-reperfusion injury has been shown to reduce NET formation and result in long-term benefit in heart function (50). NETs have also been identified in patients with ST-elevation myocardial infarction (STEMI) (51). Glycolysis and the glucose transporter (*Glut-1)* have also been implicated in NET formation (52). The pentose phosphate pathway (PPP) is also critical for NET release. Glucose diversion toward the PPP allows the production of nicotinamide adenine dinucleotide phosphate (NADPH), which in turn fuels NADPH oxidase to produce superoxide, and NETs release (53).

### In Addition to Their Pro-Inflammatory Roles, Neutrophils and Their Metabolism Also Play a Role in Inflammation Resolution

For example, neutrophils metabolize the specialized pro-resolving lipid Resolvin E1 (RvE1), derived from omega-3 eicosapentaenoic acid, into inactive byproducts. This may be part of a coordinated return to homeostasis as RvE1 is a proresolving lipid mediator that is pro-phagocytic at low nM concentrations (54). Neutrophils at steady state also facilitate a diurnal IL-23 signaling axis to alert the need for additional myeloid cells (55). During inflammation, neutrophils recruit inflammatory monocytes and macrophages (56). Inflammatory neutrophils secrete chemoattractants such as heparin binding protein (HBP) (57) and LL-37 (58) that regulate monocyte and macrophage infiltration. In heart, neutrophils trigger macrophage polarization toward an anti-inflammatory and a reparative phenotype (26), whereas depleting neutrophils with monoclonal Ly-6g antibodies in mice inhibited Ly-6Chi monocyte release from splenic reservoirs. This in turn increased macrophage proliferation in the infarct, and increased cardiomyocyte death, fibrosis, and markers of heart failure. An interesting question to answer is whether neutrophils may also instruct cardiac macrophage reprogramming through rewired cellular metabolism. Phagocytic uptake of dying neutrophils and apoptotic cells is known to induce an anti-inflammatory response. The phagocytic program is regulated by nuclear receptors including LXR (59), PPARγ (60), and PPARδ, which are known to be master-regulators of lipid and mitochondrial metabolism (61).

## Macrophage Metabolism and Links to Cardiac Repair

### For Decades we Have Appreciated That Activated Macrophages Often Exhibit Features of Heightened Glycolysis and Warburg Metabolism (62)

This phenotype is characterized by augmented glycolysis, increased expression of glycolytic enzymes (63), and diminished oxygen consumption. Although a less efficient path for the generation of ATP, glycolysis does not requires oxygen, which is rate limiting during myocardial hypoxia. Oxygen reductions stabilize macrophage HIFs (64) and accumulation of the HIF-1α isoform is linked to glycolytic induction, including glucose transporter GLUT-1 (65), hexokinase (66), and 6-phosphofructokinase (67). Simultaneously, HIF-1α may antagonize oxidative phosphorylation by shunting pyruvate away from the mitochondria through the action of PDK1 (68). Interestingly, HIFs also sense changes in metabolism from extracellular sources. For example, lactic acid produced by glycolytic tumor cells, induces a HIF-1α-dependent polarization of tumor-associated macrophages (69). In macrophages, metabolic regulation by HIFs appears to be a property specific to the HIF-1α isoform. HIF-2α in contrast induces inflammatory cytokines, independent of significant changes in glycolysis (70). During myocardial infarction, *Hif-1*α deficiency in myeloid cells decreased leukocyte recruitment inside the damaged tissue and improved cardiac function post MI (71). Glycolysis and HIFs are also triggered by the activation of macrophage toll like receptors (72). In heart, liberation of endogenous damage-associated molecular patterns (DAMPs) become ligands for TLR activation (73). TLR4-activation stimulates glycolysis and triggers pyruvate kinase-M2, in cooperation with HIF-1α and to transactivate the expression of the *Il1*β gene (74). As discussed further below, HIF-1α is activated after TLR4 stimulation through the accumulation of TCA-derived succinate (75). Under aerobic conditions, AKT-mTOR signaling activates HIF-1α to “train” immunity (76) through epigenetic remodeling. Trained immune cells are also characterized by a high ratio of nicotinamide adenine dinucleotide (NAD+) relative to its reduced form NADH. NAD+ has been implicated in key macrophage signaling events. For example, NAD+ depletion can induce macrophage necroptosis while NAD+ replenishment protects cell from necroptosis and alleviates cytotoxicity (77). In a therapeutic context, intraperitoneal injection of NAD precursor nicotinamide mononucleotide (NMN) significantly increased NAD+ in heart and protected from I/R injury (78).

The propensity for glycolytic activation in innate immune cells during inflammation is exploited in the clinic. For example, glucose analog 2-(18F)-fluoro-2-deoxy-D-glucose (FDG) concentrates in tissue with high glycolytic activity (79), including organs rich in inflammatory macrophages (80); this is readily detected by positron emission tomography. Granulocyte-macrophage colony-stimulating factor (GM-CSF), which attracts phagocytes to the heart (81), enhances macrophage glycolytic activity and 18F-FDG-update during inflammation *in vivo* (82), and after MI (83). More recent approaches have non-invasively imaged glycolytic cardiac inflammation with hyperpolarized magnetic resonance through employ of [1-^13^C] pyruvate and lactate (84). Development of similar clinical techniques to monitor the balance between immune cell glycolysis and oxidative phosphorylation may be useful in differentiating discreet stages of inflammation and inflammation resolution.

#### The PPP

The switch to glycolysis from oxidative phosphorylation is more efficient for the generation of biosynthetic intermediates that may fuel macrophages in the response to tissue injury. This is in part due to the pentose phosphate pathway (PPP), or hexose monophosphate shunt. Increased glycolytic utilization drives buildup of metabolites like glucose-6-phosphate, which flux into the PPP. This shunt is an alternative glucose-oxidizing pathway that is essential for the production of purines and pyrimidines for nucleic acid synthesis. The PPP also produces NADPH through Glucose-6-phosphate dehydrogenase (G6PD) and phosphogluconate dehydrogenase (PGD). NADPH is fed into NADPH oxidase and NOX2 on phagosomes (85, 86) to generate localized reactive oxygen species; this may also be useful for the breakdown of engulfed apoptotic bodies during transport to the lysosome. NADPH is additionally important during light-chain 3 associated phagocytosis (87). Thus, the PPP may serve as an important metabolic shunt in phagocytes during early phagocytic phases of MI. Interestingly, metabolite profiling of blood from patients undergoing planned MI, revealed a signature that included alterations in the PPP (88). Due to its dual role, the PPP is also essential to protect macrophages against oxidation by fueling with numerous NADPH-dependent antioxidative enzymes as glutathione-disulfide reductase (89). In the case of excess of circulating glucose, as in diabetic patients, G6PD, a rate-limiting enzyme of the PPP, may be reduced. This can elevate oxidative stress and cell death susceptibility (90). Hyperglycemia promotes myelopoiesis, increases circulating neutrophils, and impairs inflammation resolution in atherosclerosis (91). Elevated myelopoiesis is also a risk factor for impaired cardiac healing post MI (92). Moreover, hyperglycemia leads to increased advanced glycation end products and inflammatory macrophage RAGE signaling (93); this may be targeted by RAGE antagonists (93). The consequences of insulin resistance on macrophage immunometabolic function requires further study.

### Glycolytic Pathways Also Feed Into the Mitochondria

Beyond their energetic function, mitochondria act as signaling organelles (94). This was prominently exemplified by the discovery that mitochondrial cytochrome c is released into the cytosol to activate cell death signaling (95). Mitochondria also release metabolites and reactive oxygen species (96) which activate cytosolic signaling. Signaling complexes may also form on outer mitochondrial membranes and form connections with the endoplasmic reticulum as mitochondria-associated membranes and transport lipids between organelles (97).

In the case of inflammation, toll like receptor signaling triggers re-localization of mitochondria to sites proximal to phagosomes. This subcellular trafficking augments the production of mitochondrial reactive oxygen species (ROS) (98).

ROS is also generated by the mitochondrial electron transport chain/ETC. The ETC is composed of four respiratory complexes. Complex I, III, and IV have been found to assemble as larger molecular super-complexes (99). Interestingly, TLR signaling and inflammasome NLRP3 activation reduce the formation of these super-complexes, in turn leading to increased CII activity. The adaptation of ETC complex assembly, including CII activity, is linked to macrophage activation (100). The ETC is also tightly coupled to the TCA. During inflammation, integrated metabolomics and transcriptomics analyses have revealed TCA cycle breaks. For example, one disruption occurs at isocitrate dehydrogenase (IDH) (101). This IDH break leads to the escalation of itaconate (102), which is highly produced by activated macrophages (103). Itaconate in activated macrophages inhibits dehydrogenase-mediated oxidation of succinate and is anti-inflammatory in culture as well as after myocardial ischemia-reperfusion injury (102). As introduced above, a second inflammation-induced TCA break may occur at succinate (101). Succinate is linked to macrophage activation through the activity of succinate dehydrogenase (104). Accumulation of succinate can be transported from the mitochondria to cytosol where in excess it impairs the activity of Prolyl hydroxylases (PHDs), which in turn leads to HIF-1α stabilization and pro-inflammatory IL-1β production (74, 104). Accumulation of succinate from the tricarboxylic acid cycle contributes to activation of the NLRP3 (NOD-like receptor family, pyrin domain containing) inflammasome (105) to activate IL-1β. Succinate also contributes to mitochondrial ROS (mROS) formation. mROS exacerbates issue injury after ischemia-reperfusion (106). Accumulations in succinate may be reoxidized by succinate dehydrogenase upon reperfusion, driving extensive ROS generation by reverse electron transport at mitochondrial complex I (104). Decreasing ischemic succinate accumulation is sufficient to ameliorate ischemia reperfusion injury in murine models of heart attacks (107).

Accumulating evidence highlights the importance of mitochondrial dysfunction in inflammation and cardiovascular disease. In macrophages, mitochondrial dysfunction prevents repolarization of inflammatory macrophages (108), and the NLRP3 inflammasome senses mitochondrial dysfunction (105). Recent findings provide evidence for mitochondrial DNA damage and decreased activity of mitochondrial electron transport complexes (109) and mitochondrial respiration (110) in coronary artery disease; restoring mitochondrial DNA copy number in these settings may be ameliorative (111). Mitochondrial dysfunction is characterized by uncoupling of the electron transport chain, reduced production of adenosine triphosphate, and elevated production of ROS. Excessive mROS may do damage to mitochondrial DNA. Mitochondrial DNA damage and dysfunction is also associated with oxidative stress post MI. For example, compared to control, the mtDNA-encoded gene transcripts of CI and CIII of the electron transport chain are decreased, along with the enzyme activity of complexes I, III, and IV after MI (112). Ultimately, dysfunctional mitochondria may be cleared or metabolized through mitophagy.

### Mitochondria in Anti-inflammatory Processes

In comparison to initial links between glycolytic metabolism and pro-inflammatory macrophage activation, our understanding of metabolic integration with anti-inflammatory macrophage polarization has trailed behind, yet has made significant recent strides forward. During aerobic respiration, glycolytic pyruvate is shuttled to the mitochondria for entry into the enzyme-catalyzed reactions of the tricarboxylic acid cycle (113). In studies that employed 2-deoxy-D-glucose (2DG) to competitively inhibit the production of glucose-6-phosphate from glucose, glycolysis was implicated in generation of pyruvate for entry into the TCA cycle, as well as regulation of interleukin-4 dependent oxidative phosphorylation (114). In addition, glycolysis may contribute to alternative macrophage activation via an M-CSF-induced mTORC2-IR4 axis, acting in parallel with IL-4Rα/Stat6 signaling (114). However, a recent study concluded that glycolysis is not required for alternative activation of macrophages. The differences between the studies may be explained by off-target effects of 2-DG on oxidative phosphorylation and the TCA cycle (115). Prior studies associated cell-intrinsic lipolysis and fatty acid oxidation to IL-4 induced macrophage polarization by etomoxir (ETO), an inhibitor of carnitine palmitoyl transferase (CPT1) (116). Subsequent studies in *Cpt1a-deficient* macrophages did not reveal contributions of LC-fatty acid oxidation during alternative macrophage polarization. Rather, etomoxir was found to have the potential to reduce the pool of Coenzyme A (CoA) during IL-4-triggerd macrophage polarization (117). Furthermore, *Cpt-2-deficient* macrophages also did not show impaired macrophage polarization, raising further questions on the role of long chain fatty acid oxidation (LC-FAO) and macrophage polarization (118). Nevertheless, inhibition of mitochondrial oxidative phosphorylation has been shown to prevent repolarization from classically activated to alternatively activated macrophages. For example, inhibiting nitric oxide production in classically activated macrophages dampens declines in mitochondrial function and facilitates repolarization to the alternative state (108). IL4 may have potential post MI in promoting reparative phase cardiac macrophages (119).

### Mitochondrial Metabolism and Epigenetic Modulation

Epigenetics play a key role in the establishment of the so called M1 and M2 program. α-KG produced by the Krebs cycle, or generated from glutaminolysis, is an important co-factor for numerous enzymes involved in epigenetic modification of DNA and histones (120). Among these enzymes, *Jmjd3*, an essential H3K27 demethylase, has been reported to promote M2 activation whereas its activity attenuates inflammation in classically activated macrophages. In M2 macrophages, α-KG accumulates whereas its abundance is decreased in M1 macrophages due to its broken Krebs cycle, as well as a higher α-KG dehydrogenase activity to the benefit of succinate accumulation (102). Interestingly, α-KG/succinate ratio can modulate *Jmjd3* activity (121). Thus, manipulation of this ratio may allow the control of macrophage polarization state. Another example of the epigenetic control over macrophage inflammatory status is given by TET2, a deoxyribonucleic acid (DNA) methyltransferase that is highly expressed in macrophages. TET2 restricts inflammatory gene activation through DNA methylation at 5-methylcytosine sites, whereas its deletion increases inflammation at basal levels and after LPS stimulation (122). Interestingly, in experimental mice harboring *Tet2* deletion, myeloid *Tet2* deficiency accelerated heart failure through the IL-1β/NLRP3 inflammasome (123). Clonal hematopoiesis associated with *Tet2*-deficiency has been shown to accelerate atherosclerosis in experimental mice (124, 125), whereas whole-exome sequencing of carriers of clonal hematopoiesis of indeterminate potential (CHIP) patients indicated that mutations in TET2 are associated with heightened coronary heart disease risk (125).

### Clearance and Catabolism of Dying Cardiomyocytes and Apoptotic Cells by Efferocytosis (126) Is Necessary for Cardiac Repair (127, 128)

Efferocytosis is distinct mechanistically and metabolically to the substrates acquired during microbial phagocytosis. The engulfment of dying cells has the capacity to double the phagocyte cellular content and provide new nutrient substrates for signaling reactions. Early studies connected the cellular metabolism of efferocytosis to generation of high energy phosphates which are necessary for actin-mediated engulfment of apoptotic bodies (45). Later studies elucidated roles for mitochondrial uncoupling proteins in regulating continued dying cell engulfment (129). Also, mitochondrial fission machinery (130) is necessary for multiple rounds of efferocytosis, which is likely critical to tissue injury that is characterized by heightened cell turnover. These later scenarios suggest conserved relationships and feedback mechanisms between mitochondrial metabolic sensing and uptake of dying cells. In the case of myocardial infarction and after ischemic reperfusion, dying cardiomyocytes are engulfed by recruited and resident cardiac macrophages (128, 131); this correlates directly with both heightened macrophage oxygen consumption and anti-inflammatory *Il10* cytokine expression. IL-10 is a hallmark cytokine produced in disparate settings of tissue injury. Within the re-perfused myocardium, IL-10 is produced and linked to the regulation of extracellular matrix biosynthesis (132) and cardiac repair (133). IL10 is linked to inflammation resolution and may inhibit toll like receptor-associated glucose uptake through mTOR and induce macrophage polarization toward oxidative phosphorylation (134). Consistent with these relationships, macrophages fed apoptotic cells exhibit a transcriptional and metabolic signature polarized for fatty acid oxidation, and mitochondrial organelles can be found in close proximity to phagosomes. So how might mitochondrial metabolism be linked to anti-inflammatory cytokine production? One answer may be NAD. The citric acid cycle generates NADH reducing equivalents which are fed into the electron transport chain for oxidative phosphorylation (113). Efferocytosis increases NAD+, and NAD is sufficient to for efferocytic-induced IL10 through the activation of Sirtuin-mediated signaling (135). In comparison, glucose availability is linked to both lower cytosolic NADH:NAD+ ratios and reduced NF-kB activation, and macrophage pro-inflammatory gene expression. This can be rescued by forced elevation of NADH, or reduced expression of the NADH-sensitive transcriptional co-repressor *CtBP* (136). Efferocytosis also triggers phagocyte polarization and metabolic parallels and distinctions may be made between efferocytic-induced polarization vs. cytokine-induced. For example, alternative polarization of macrophages by interleukin-4 (IL-4) has also been linked to the mobilization of intra- and extra-cellular free fatty acids (FFAs). However, unlike after IL-4, glycolytic requirements may be unnecessary for efferocytic-induced IL10.

An understanding of these basic mechanisms of efferocytic metabolism is important in the context that cardiovascular disease is associated with impaired phagocytosis (137, 138) and metabolic imbalance. For example, obesity is linked to defective efferocytosis in association with elevated levels of saturated fatty acids. This may be corrected by the action of omega-3 fatty acids (139). Impairments of cholesterol efflux from macrophages under conditions of excess lipid, as occurs during cardiovascular disease, may lead to macrophage death (140). Interestingly, lysosomal cholesterol hydrolysis couples efferocytosis to anti-inflammatory oxysterol production (141). Thus, cellular imbalances of metabolites during disease could in principle dysregulate the integrated metabolic response of macrophages to promote tissue repair.

### Amino Acids

Amino acids are utilized for numerous anabolic reactions in macrophages and are sensed by mammalian target of the rapamycin (mTOR) (142). mTOR-inhibitors such as rapamycin block immune cell activation and can inhibit allograft rejection. mTOR is particularly sensitive to branched-chain amino acids/BCAAs (143). The BCAA isoleucine is required for cell proliferation in a mTORC1-dependent pathway. For example, experimental mice fed a BCAA-reduced diet exhibited decreased numbers of Foxp3^+^ T-regulatory cells (144). BCAA catabolism is initiated by branched-chain aminotransferase (BCAT) enzymes. BCATs transfer α-amino groups from BCAAs to αKG and BCAT1 deficiency can lead to alpha-KG accumulation (145). Furthermore, selective inhibition of BCAT1 activity by the compound ERG240 leads to decreased oxygen consumption and glycolysis through the down regulation of IRG1 expression, which leads to decrease itaconate production in human macrophages. ERG240 has also been shown to reduce macrophage migration and inflammatory responses in immune-mediated inflammation (146). Further studies are warranted to test this pathway in disparate inflammatory settings of inflammation, including heart.

One of the earliest links between amino acids and phagocytes was the discovery that arginine is important to macrophage function. Classically activated macrophages metabolize L-arginine through inducible nitric oxide synthase (147) whereas alternatively activated macrophages metabolize L-arginine trough arginase-1 (Arg1) (148). Macrophages that are stimulated with TLR ligands express nitric oxide synthase, which metabolizes arginine to nitric oxide and citrulline (149). Extracellular arginine supports the initial burst of NO, which is also required for control of infection (150). In comparison, alternatively activated macrophages express Arg1 that hydrolyzes arginine to ornithine and urea. In this setting, *Arg1-deficiency* in macrophages leads to increased pro-inflammatory macrophage polarization (151). In heart, L-arginine is associated with a higher risk of ischemic heart disease, and L-arginine administration post-infarction may be associated with a higher post-infarction mortality (152). Interestingly, oral administration of arginine improved wound healing and increased insulin-like growth factor-1 (IGF1) in post-surgery patients (153). Also, inhibition of Arg1 activity did not alter alternatively activated macrophage numbers but increased local inflammation and defects in matrix deposition (154).

### Glutamine

may also be important in proliferating immune cells as an alternative input for the TCA cycle, or as a source of citrate for fatty acid synthesis. For example, glutamine has recently been implicated in T-cell metabolism (155). In phagocytes, glutamine metabolism provides support for macrophage activation. Multiple polarization states of macrophages rely on glutamine to mount desirable immune functions. For example, classically activated macrophages exhibit increased glucose and glutamine consumption and suppress oxidative metabolism (156). In IL-4 activated macrophages, the TCA cycle is intact and glutamine is metabolized into UDP-GlcNAc, which is important for glycosylation of lectin or mannose receptors for pathogen recognition. Glutamine deprivation reduces this macrophage polarization (101). Later studies reported that glutamine supports alternative macrophage activation through suppressing NFκB-dependent classical macrophage activation. αKG generated through glutaminolysis is required for PHD-dependent proline hydroxylation of IKKβ, thus suppressing NF-κB downstream of LPS. *In vivo*, glutamine metabolism supports induction of endotoxin tolerance in an αKG dependent manner (121). Glutamine metabolism has also been shown to be increased in trained monocytes. For example, glutaminolysis led to replenishment of the TCA cycle and accumulation of fumarate. Fumarate induced monocyte epigenetic reprogramming by inhibiting KDM5 histone demethylases. Inhibiting glutaminolysis and cholesterol metabolism inhibited blocked trained immunity in monocytes (157). Moreover, glutamine deficiency reduced lipid-induced lysosome dysfunction, mTORC1 activity, and thereby increased autophagy and reduced cell death in macrophages (158). During ischemic heart disease, glutamine enhances recovery from acute ischemia in isolated rate hearts. Furthermore, post-ischemic reperfusion of isolated rat hearts with glutamine resulted in a full recovery of cardiac output (159).

## Summary and Future Research

The field of immunometabolism arose to integrate cellular nutrient metabolism with innate and adaptive immune cell function. Largely absent are past preconceived notions that cellular nutrient processing is solely for energetic currency. Nutritional status has long been associated with optimal wound healing, yet early molecular interest grew in part from pioneering recognition of the association between excess metabolism and cell stress (160). During tissue injury, the integration of information between nutrient availability and anabolic needs leads cells to adapt their metabolism to ensure their functions.

In the translational and clinical arena, current interest of relationships between metabolism and inflammation is high. Compounds that were initially prioritized for metabolic benefit, also act through immunometabolic nodes. For example, the insulin sensitizer metformin (dimethylbiguanide) has effects beyond glucose control. Metformin stimulates the energy sensor AMP-activated protein kinase (AMPK), which is activated during nutrient deficiency (161). Induction of AMPK elevates the fatty acid transporter CPT1 and drives β-oxidation. Simultaneously, AMPK may antagonize inflammatory NFκB gene expression and has been implicated in anti-inflammatory macrophages through an axis of SIRT1-PGC-1α mitochondrial biogenesis (162). AMPK also inhibits the glycolytic-inhibitor mTOR (163), and mTOR-inhibitor rapamycin has been shown to attenuate myocardial damage after ischemia reperfusion (164). Separately, inhibitors of the sodium-glucose cotransporter SGLT2 may have promise in cardiovascular outcomes (165), yet their action on macrophages are unclear and caution must be taken given potential side effects of urinary tract infections. In comparison, drugs that directly target inflammatory cytokines, may also regulate cellular metabolism. For instance, IL-1β is notable as introduced above for its sensitivity and responsiveness to metabolic alterations (166). Another case in point is the finding that elevated glucose levels after feeding induces macrophage IL-1β secretion (167). In the case of myocardial infarction (MI), IL-1β is markedly upregulated in infarcted myocardium (168) and in blood (169), paralleling peripheral cell blood counts. Preclinical studies have shown that anti-IL-1β therapy reduced post MI hematopoiesis and leukocytosis, enhanced inflammation resolution in the infarct, and ameliorated post-MI heart failure (170). The landmark Canakinumab Anti-inflammatory Thrombosis Outcome Study (CANTOS) inhibited interleukin 1β (IL-1B) in survivors of myocardial infarction and reduced cardiovascular events (171, 172). However, IL-1β exerts both detrimental and beneficial effects (173). Thus, further studies are warranted to optimize the full potential of IL-1β targeting in heart, as well as potential immunometabolic consequences (174). This is particularly the case in the face of recent negative clinical results after treatment with low dose methotrexate (175).

Because of the complicated integration and flux of numerous metabolic pathways, an informed future perspective will benefit from a coupled global network analysis of metabolomics and inflammatory signaling axes. As an example, integrated top-down transcriptional and metabolic omics approaches were key in revealing that pro-inflammatory macrophages exhibit a disrupted TCA cycle (101). These global and non-biased methods are necessary to completely appreciate the heterogeneity of a diverse phagocyte population. Insight into the metabolic activity of single cells holds the potential to uncover nuanced degrees of immunometabolic control, that we do not currently comprehend. In combination with genetic lineage tracing, fate mapping, and epigenetics signatures, we will also better understand how metabolism may regulate immune cell origins and their differentiation during inflammation (176). Surely, we have only scratched the surface of the spectrum of metabolites that are protagonists of signal transduction in immune cells and heart.

Finally, much of our understanding of immunometabolism has originated from studies in cell culture and experimental rodents. Therefore, it will be important to test the conservation of these findings in human cells and in the clinic. These are important considerations given that mice naturally exhibit a basal metabolic rate per gram of body weight that is greater than that of humans. Advancements and accessibility of induced pluripotent stem cells that may be differentiated into immune cells (177) will facilitate human-mouse comparisons, as well as genetic associations. In experimental animals, multiple studies that have targeted inflammatory pathways have shown promise in enhancing cardiac repair, however, a disconnect exists between translating outcomes in preclinical models to improved patient outcomes (178). This is a challenge to understand experimental, species, and clinical distinctions between independent studies. It is also a challenge to dig deeper into fundamental mechanisms that regulate immunometabolic signaling in cardiovascular disease.

## Author Contributions

All authors listed have made a substantial, direct and intellectual contribution to the work, and approved it for publication.

### Conflict of Interest Statement

The authors declare that the research was conducted in the absence of any commercial or financial relationships that could be construed as a potential conflict of interest.

## References

[1] Benjamin EJ Virani SS Callaway CW Chamberlain AM Chang AR Cheng S. Heart disease and stroke statistics-2018 update: a report from the American Heart Association. Circulation. (2018) 137:e67–e492. 10.1161/CIR.0000000000000558 29386200

[2] Liang CS Delehanty JD. Increasing post-myocardial infarction heart failure incidence in elderly patients a call for action. J Am Coll Cardiol. (2009) 53:21–3. 10.1016/j.jacc.2008.09.026 19118719

[3] Hausenloy DJ Yellon DM. Myocardial ischemia-reperfusion injury: a neglected therapeutic target. J Clin Invest. (2013) 123:92–100. 10.1172/JCI62874 23281415 PMC3533275

[4] Hansson GK Stemme S Geng YJ Holm J. Can immunocompetent cells and their cytokines play a role in atherogenesis? Nouvelle Rev Francaise d'hematol. (1992) 34(Suppl):S43–46. 1340528

[5] Libby P Clinton SK. Cytokines as mediators of vascular pathology. Nouvelle Rev Francaise d'hematol. (1992) 34(Suppl):S47–53. 1340529

[6] Serhan CN Savill J. Resolution of inflammation: the beginning programs the end. Nat Immunol. (2005) 6:1191–7. 10.1038/ni1276 16369558

[7] Yvan-Charvet L Pagler T Gautier EL Avagyan S Siry RL Han S. ATP-binding cassette transporters and HDL suppress hematopoietic stem cell proliferation. Science. (2010) 328:1689–93. 10.1126/science.1189731 20488992 PMC3032591

[8] Panizzi P Swirski FK Figueiredo JL Waterman P Sosnovik DE Aikawa E. Impaired infarct healing in atherosclerotic mice with Ly-6C(hi) monocytosis. J Am Coll Cardiol. (2010) 55:1629–38. 10.1016/j.jacc.2009.08.089 20378083 PMC2852892

[9] Dutta P Courties G Wei Y Leuschner F Gorbatov R Robbins CS. Myocardial infarction accelerates atherosclerosis. Nature. (2012) 487:325–9. 10.1038/nature11260 22763456 PMC3401326

[10] Swirski FK Nahrendorf M Etzrodt M Wildgruber M Cortez-Retamozo V Panizzi P. Identification of splenic reservoir monocytes and their deployment to inflammatory sites. Science. (2009) 325:612–6. 10.1126/science.1175202 19644120 PMC2803111

[11] Nahrendorf M Swirski FK. Monocyte and macrophage heterogeneity in the heart. Circ Res. (2013) 112:1624–33. 10.1161/CIRCRESAHA.113.300890 23743228 PMC3753681

[12] Frangogiannis NG Smith CW Entman ML. The inflammatory response in myocardial infarction. Cardiovasc Res. (2002) 53:31–47. 10.1016/S0008-6363(01)00434-5 11744011

[13] Whelan RS Kaplinskiy V Kitsis RN. Cell death in the pathogenesis of heart disease: mechanisms and significance. Annu Rev Physiol. (2010) 72:19–44. 10.1146/annurev.physiol.010908.163111 20148665 PMC12973270

[14] Chen P Zuo H Xiong H Kolar MJ Chu Q Saghatelian A. Gpr132 sensing of lactate mediates tumor-macrophage interplay to promote breast cancer metastasis. Proc Natl Acad Sci USA. (2017) 114:580–5. 10.1073/pnas.1614035114 28049847 PMC5255630

[15] Krysko DV Agostinis P Krysko O Garg AD Bachert C Lambrecht BN. Emerging role of damage-associated molecular patterns derived from mitochondria in inflammation. Trends Immunol. (2011) 32:157–64. 10.1016/j.it.2011.01.005 21334975

[16] Deniset JF Kubes P. Recent advances in understanding neutrophils. F1000Research. (2016) 5:2912. 10.12688/f1000research.9691.1 28105328 PMC5225409

[17] Meissner J Irfan A Twerenbold R Mueller S Reiter M Haaf P. Use of neutrophil count in early diagnosis and risk stratification of AMI. Am J Med. (2011) 124:534–42. 10.1016/j.amjmed.2010.10.023 21507368

[18] Soo A Maher B McCarthy J Nolke L Wood A Watson RW. Pre-operative determination of an individual's neutrophil response: a potential predictor of early cardiac transplant cellular rejection. J Heart Lung Transplant. (2009) 28:1198–205. 10.1016/j.healun.2009.05.026 19782611

[19] Dragu R Khoury S Zuckerman R Suleiman M Mutlak D Agmon Y. Predictive value of white blood cell subtypes for long-term outcome following myocardial infarction. Atherosclerosis. (2008) 196:405–12. 10.1016/j.atherosclerosis.2006.11.022 17173924

[20] Chia S Nagurney JT Brown DF Raffel OC Bamberg F Senatore F. Association of leukocyte and neutrophil counts with infarct size, left ventricular function and outcomes after percutaneous coronary intervention for ST-elevation myocardial infarction. Am J Cardiol. (2009) 103:333–7. 10.1016/j.amjcard.2008.09.085 19166685

[21] Ali M Pulli B Courties G Tricot B Sebas M Iwamoto Y. Myeloperoxidase inhibition improves ventricular function and remodeling after experimental myocardial infarction. JACC Basic Trans Sci. (2016) 1:633–43. 10.1016/j.jacbts.2016.09.004 30167547 PMC6113523

[22] Romson JL Hook BG Kunkel SL Abrams GD Schork MA Lucchesi BR. Reduction of the extent of ischemic myocardial injury by neutrophil depletion in the dog. Circulation. (1983) 67:1016–23. 10.1161/01.CIR.67.5.1016 6831665

[23] Nijhuis J Rensen SS Slaats Y van Dielen FM Buurman WA Greve JW. Neutrophil activation in morbid obesity, chronic activation of acute inflammation. Obesity. (2009) 17:2014–8. 10.1038/oby.2009.113 19390527

[24] Mazor R Shurtz-Swirski R Farah R Kristal B Shapiro G Dorlechter F. Primed polymorphonuclear leukocytes constitute a possible link between inflammation and oxidative stress in hyperlipidemic patients. Atherosclerosis. (2008) 197:937–43. 10.1016/j.atherosclerosis.2007.08.014 17869258

[25] Wong SL Demers M Martinod K Gallant M Wang Y Goldfine AB. Diabetes primes neutrophils to undergo NETosis, which impairs wound healing. Nat Med. (2015) 21:815–9. 10.1038/nm.3887 26076037 PMC4631120

[26] Horckmans M Ring L Duchene J Santovito D Schloss MJ Drechsler M. Neutrophils orchestrate post-myocardial infarction healing by polarizing macrophages towards a reparative phenotype. Eur Heart J. (2017) 38:187–97. 10.1093/eurheartj/ehw002 28158426

[27] Ma Y Yabluchanskiy A Iyer RP Cannon PL Flynn ER Jung M. Temporal neutrophil polarization following myocardial infarction. Cardiovasc Res. (2016) 110:51–61. 10.1093/cvr/cvw024 26825554 PMC4798046

[28] Fridlender ZG Sun J Kim S Kapoor V Cheng G Ling L. Polarization of tumor-associated neutrophil phenotype by TGF-beta: “N1” versus “N2” TAN. Cancer Cell. (2009) 16:183–94. 10.1016/j.ccr.2009.06.017 19732719 PMC2754404

[29] Early JO Curtis AM. Immunometabolism: is it under the eye of the clock? Semin Immunol. (2016) 28:478–90. 10.1016/j.smim.2016.10.006 27884543

[30] Dallmann R Viola AU Tarokh L Cajochen C Brown SA. The human circadian metabolome. Proc Natl Acad Sci USA. (2012) 109:2625–9. 10.1073/pnas.1114410109 22308371 PMC3289302

[31] Eckel-Mahan KL Patel VR Mohney RP Vignola KS Baldi P Sassone-Corsi P. Coordination of the transcriptome and metabolome by the circadian clock. Proc Natl Acad Sci USA. (2012) 109:5541–6. 10.1073/pnas.1118726109 22431615 PMC3325727

[32] Hong HK Maury E Ramsey KM Perelis M Marcheva B Omura C. Requirement for NF-kappaB in maintenance of molecular and behavioral circadian rhythms in mice. Genes Dev. (2018) 32:1367–79. 10.1101/gad.319228.118 30366905 PMC6217733

[33] Casanova-Acebes M Pitaval C Weiss LA Nombela-Arrieta C Chevre RN. Rhythmic modulation of the hematopoietic niche through neutrophil clearance. Cell. (2013) 153:1025–35. 10.1016/j.cell.2013.04.040 23706740 PMC4128329

[34] Schloss MJ Horckmans M Nitz K Duchene J Drechsler M Bidzhekov K. The time-of-day of myocardial infarction onset affects healing through oscillations in cardiac neutrophil recruitment. EMBO Mol Med. (2016) 8:937–48. 10.15252/emmm.201506083 27226028 PMC4967945

[35] Kalsbeek A Yi CX La Fleur SE Fliers E. The hypothalamic clock and its control of glucose homeostasis. Trends Endocrinol Metabol. (2010) 21:402–10. 10.1016/j.tem.2010.02.005 20303779

[36] Scheiermann C Kunisaki Y Lucas D Chow A Jang JE Zhang D. Adrenergic nerves govern circadian leukocyte recruitment to tissues. Immunity. (2012) 37:290–301. 10.1016/j.immuni.2012.05.021 22863835 PMC3428436

[37] Ella K Csepanyi-Komi R Kaldi K. Circadian regulation of human peripheral neutrophils. Brain Behav Immun. (2016) 57:209–21. 10.1016/j.bbi.2016.04.016 27132055

[38] Peek CB Affinati AH Ramsey KM Kuo HY Yu W Sena LA. Circadian clock NAD+ cycle drives mitochondrial oxidative metabolism in mice. Science. (2013) 342:1243417. 10.1126/science.1243417 24051248 PMC3963134

[39] Gaul DS Weber J van Tits LJ Sluka S Pasterk L Reiner MF. Loss of Sirt3 accelerates arterial thrombosis by increasing formation of neutrophil extracellular traps and plasma tissue factor activity. Cardiovasc Res. (2018) 114:1178–88. 10.1093/cvr/cvy036 29444200 PMC6014146

[40] Riffelmacher T Clarke A Richter FC Stranks A Pandey S Danielli S. Autophagy-dependent generation of free fatty acids is critical for normal neutrophil differentiation. Immunity. (2017) 47:466–480.e465. 10.1016/j.immuni.2017.08.005 28916263 PMC5610174

[41] Rozman S Yousefi S Oberson K Kaufmann T Benarafa C Simon HU. The generation of neutrophils in the bone marrow is controlled by autophagy. Cell Death Differ. (2015) 22:445–56. 10.1038/cdd.2014.169 25323583 PMC4326574

[42] Tanimura A Miyoshi K Horiguchi T Hagita H Fujisawa K Noma T. Mitochondrial activity and unfolded protein response are required for neutrophil differentiation. Cell Physiol Biochem. (2018) 47:1936–50. 10.1159/000491464 29972819

[43] Grenda DS Murakami M Ghatak J Xia J Boxer LA Dale D. Mutations of the ELA2 gene found in patients with severe congenital neutropenia induce the unfolded protein response and cellular apoptosis. Blood. (2007) 110:4179–87. 10.1182/blood-2006-11-057299 17761833 PMC2234798

[44] Kollner I Sodeik B Schreek S Heyn H von Neuhoff N Germeshausen M. Mutations in neutrophil elastase causing congenital neutropenia lead to cytoplasmic protein accumulation and induction of the unfolded protein response. Blood. (2006) 108:493–500. 10.1182/blood-2005-11-4689 16551967

[45] Oren R Farnham AE Saito K Milofsky E Karnovsky ML. Metabolic patterns in three types of phagocytizing cells. J Cell Biol. (1963) 17:487–501. 10.1083/jcb.17.3.487 13940299 PMC2106210

[46] Hirsch JG Fedorko ME. Ultrastructure of human leukocytes after simultaneous fixation with glutaraldehyde and osmium tetroxide and “postfixation” in uranyl acetate. J Cell Biol. (1968) 38:615–27. 10.1083/jcb.38.3.615 4874495 PMC2108377

[47] Maianski NA Geissler J Srinivasula SM Alnemri ES Roos D Kuijpers TW. Functional characterization of mitochondria in neutrophils: a role restricted to apoptosis. Cell Death Differ. (2004) 11:143–53. 10.1038/sj.cdd.4401320 14576767

[48] Fossati G Moulding DA Spiller DG Moots RJ White MR Edwards SW. The mitochondrial network of human neutrophils: role in chemotaxis, phagocytosis, respiratory burst activation, and commitment to apoptosis. J Immunol. (2003) 170:1964–72. 10.4049/jimmunol.170.4.1964 12574365

[49] Borregaard N Herlin T. Energy metabolism of human neutrophils during phagocytosis. J Clin Invest. (1982) 70:550–7. 10.1172/JCI110647 7107894 PMC370256

[50] Ge L Zhou X Ji WJ Lu RY Zhang Y Zhang YD. Neutrophil extracellular traps in ischemia-reperfusion injury-induced myocardial no-reflow: therapeutic potential of DNase-based reperfusion strategy. Am J Physiol Heart Circ Physiol. (2015) 308:H500–9. 10.1152/ajpheart.00381.2014 25527775

[51] Helseth R Solheim S Arnesen H Seljeflot I Opstad TB The time course of markers of neutrophil extracellular traps in patients undergoing revascularisation for acute myocardial infarction or stable angina pectoris. Mediators Inflamm. (2016) 2016:2182358 10.1155/2016/2182358 28074081 PMC5198181

[52] Rodriguez-Espinosa O Rojas-Espinosa O Moreno-Altamirano MM Lopez-Villegas EO Sanchez-Garcia FJ. Metabolic requirements for neutrophil extracellular traps formation. Immunology. (2015) 145:213–24. 10.1111/imm.12437 25545227 PMC4427386

[53] Azevedo EP Rochael NC Guimaraes-Costa AB de Souza-Vieira TS Ganilho J Saraiva EM. A metabolic shift toward pentose phosphate pathway is necessary for amyloid fibril- and phorbol 12-myristate 13-acetate-induced neutrophil extracellular trap (NET) formation. J Biol Chem. (2015) 290:22174–83. 10.1074/jbc.M115.640094 26198639 PMC4571968

[54] Hong S Porter TF Lu Y Oh SF Pillai PS Serhan CN. Resolvin E1 metabolome in local inactivation during inflammation-resolution. J Immunol. (2008) 180:3512–9. 10.4049/jimmunol.180.5.3512 18292578

[55] Casanova-Acebes M Nicolas-Avila JA Li JL Garcia-Silva S Balachander A Rubio-Ponce A. Neutrophils instruct homeostatic and pathological states in naive tissues. J Exp Med. 2018:1468. (2018). 10.1084/jem.20181468 30282719 PMC6219739

[56] Soehnlein O Zernecke A Eriksson EE Rothfuchs AG Pham CT Herwald H. Neutrophil secretion products pave the way for inflammatory monocytes. Blood. (2008) 112:1461–71. 10.1182/blood-2008-02-139634 18490516 PMC3400540

[57] Soehnlein O Xie X Ulbrich H Kenne E Rotzius P Flodgaard H. Neutrophil-derived heparin-binding protein (HBP/CAP37) deposited on endothelium enhances monocyte arrest under flow conditions. J Immunol. (2005) 174:6399–405. 10.4049/jimmunol.174.10.6399 15879141

[58] De Y Chen Q Schmidt AP Anderson GM Wang JM Wooters J LL-37, the neutrophil granule- and epithelial cell-derived cathelicidin, utilizes formyl peptide receptor-like 1 (FPRL1) as a receptor to chemoattract human peripheral blood neutrophils, monocytes, and T cells. J Exp Med. (2000) 192:1069–74. 10.1084/jem.192.7.1069 11015447 PMC2193321

[59] A-Gonzalez N Bensinger SJ Hong C Beceiro S Bradley MN Zelcer N. Apoptotic cells promote their own clearance and immune tolerance through activation of the nuclear receptor LXR. Immunity. (2009) 31:245–58. 10.1016/j.immuni.2009.06.018 19646905 PMC2791787

[60] Majai G Sarang Z Csomos K Zahuczky G Fesus L. PPARgamma-dependent regulation of human macrophages in phagocytosis of apoptotic cells. Eur J Immunol. (2007) 37:1343–54. 10.1002/eji.200636398 17407194

[61] Fan W Evans R. PPARs and ERRs: molecular mediators of mitochondrial metabolism. Curr Opin Cell Biol. (2015) 33:49–54. 10.1016/j.ceb.2014.11.002 25486445 PMC4380823

[62] Hard GC. Some biochemical aspects of the immune macrophage. Br J Exp Pathol. (1970) 51:97–105. 5434449 PMC2072214

[63] Newsholme P Curi R Gordon S Newsholme EA. Metabolism of glucose, glutamine, long-chain fatty acids and ketone bodies by murine macrophages. Biochem J. (1986) 239:121–5. 10.1042/bj2390121 3800971 PMC1147248

[64] Lin N Simon MC. Hypoxia-inducible factors: key regulators of myeloid cells during inflammation. J Clin Invest. (2016) 126:3661–71. 10.1172/JCI84426 27599290 PMC5096831

[65] Chen C Pore N Behrooz A Ismail-Beigi F Maity A. Regulation of glut1 mRNA by hypoxia-inducible factor-1. Interaction between H-ras and hypoxia. J Biol Chem. (2001) 276:9519–25. 10.1074/jbc.M010144200 11120745

[66] Riddle SR Ahmad A Ahmad S Deeb SS Malkki M Schneider BK. Hypoxia induces hexokinase II gene expression in human lung cell line A549. Am J Physiol Lung Cell Mol Physiol. (2000) 278:L407–416. 10.1152/ajplung.2000.278.2.L407 10666126

[67] Minchenko A Leshchinsky I Opentanova I Sang N Srinivas V Armstead V. Hypoxia-inducible factor-1-mediated expression of the 6-phosphofructo-2-kinase/fructose-2,6-bisphosphatase-3 (PFKFB3) gene. Its possible role in the Warburg effect. J Biol Chem. (2002) 277:6183–7. 10.1074/jbc.M110978200 11744734 PMC4518871

[68] Papandreou I Cairns RA Fontana L Lim AL Denko NC. HIF-1 mediates adaptation to hypoxia by actively downregulating mitochondrial oxygen consumption. Cell Metab. (2006) 3:187–97. 10.1016/j.cmet.2006.01.012 16517406

[69] Colegio OR Chu NQ Szabo AL Chu T Rhebergen AM Jairam V. Functional polarization of tumour-associated macrophages by tumour-derived lactic acid. Nature. (2014) 513:559–63. 10.1038/nature13490 25043024 PMC4301845

[70] Imtiyaz HZ Williams EP Hickey MM Patel SA Durham AC Yuan LJ. Hypoxia-inducible factor 2alpha regulates macrophage function in mouse models of acute and tumor inflammation. J Clin Invest. (2010) 120:2699–714. 10.1172/JCI39506 20644254 PMC2912179

[71] Dong F Khalil M Kiedrowski M O'Connor C Petrovic E Zhou X. Critical role for leukocyte hypoxia inducible factor-1alpha expression in post-myocardial infarction left ventricular remodeling. Circ Res. (2010) 106:601–10. 10.1161/CIRCRESAHA.109.208967 20035082

[72] Krawczyk CM Holowka T Sun J Blagih J Amiel E DeBerardinis RJ. Toll-like receptor-induced changes in glycolytic metabolism regulate dendritic cell activation. Blood. (2010) 115:4742–9. 10.1182/blood-2009-10-249540 20351312 PMC2890190

[73] Zhang W Lavine KJ Epelman S Evans SA Weinheimer CJ Barger PM. Necrotic myocardial cells release damage-associated molecular patterns that provoke fibroblast activation *in vitro* and trigger myocardial inflammation and fibrosis *in vivo*. J Am Heart Assoc. (2015) 4:e001993. 10.1161/JAHA.115.001993 26037082 PMC4599537

[74] Tannahill GM Curtis AM Adamik J Palsson-McDermott EM McGettrick AF Goel G. Succinate is an inflammatory signal that induces IL-1beta through HIF-1alpha. Nature. (2013) 496:238–42. 10.1038/nature11986 23535595 PMC4031686

[75] Palsson-McDermott EM Curtis AM Goel G Lauterbach MA Sheedy FJ Gleeson LE Pyruvate kinase M2 regulates Hif-1alpha activity and IL-1beta induction and is a critical determinant of the warburg effect in LPS-activated macrophages. Cell Metab. (2015) 21:65–80. 10.1016/j.cmet.2014.12.005 25565206 PMC5198835

[76] Cheng SC Quintin J Cramer RA Shepardson KM Saeed S Kumar V. mTOR- and HIF-1alpha-mediated aerobic glycolysis as metabolic basis for trained immunity. Science. (2014) 345:1250684. 10.1126/science.1250684 25258083 PMC4226238

[77] Pajuelo D Gonzalez-Juarbe N Tak U Sun J Orihuela CJ Niederweis M. NAD(+) depletion triggers macrophage necroptosis, a cell death pathway exploited by *Mycobacterium tuberculosis*. Cell Rep. (2018) 24:429–40. 10.1016/j.celrep.2018.06.042 29996103 PMC6136256

[78] Yamamoto T Byun J Zhai P Ikeda Y Oka S Sadoshima J. Nicotinamide mononucleotide, an intermediate of NAD+ synthesis, protects the heart from ischemia and reperfusion. PLoS ONE. (2014) 9:e98972. 10.1371/journal.pone.0098972 24905194 PMC4048236

[79] Ahmad Sarji S Physiological uptake in FDG PET simulating disease. Biomed Imaging Interv J. (2006) 2:e59 10.2349/biij.2.4.e59 21614339 PMC3097820

[80] Zhang Z Machac J Helft G Worthley SG Tang C Zaman AG. Non-invasive imaging of atherosclerotic plaque macrophage in a rabbit model with F-18 FDG PET: a histopathological correlation. BMC Nucl Med. (2006) 6:3. 10.1186/1471-2385-6-3 16725052 PMC1479805

[81] Anzai A Choi JL He S Fenn AM Nairz M Rattik S. The infarcted myocardium solicits GM-CSF for the detrimental oversupply of inflammatory leukocytes. J Exp Med. (2017) 214:3293–310. 10.1084/jem.20170689 28978634 PMC5679174

[82] Singh P Gonzalez-Ramos S Mojena M Rosales-Mendoza CE Emami H Swanson J. GM-CSF enhances macrophage glycolytic activity *in vitro* and improves detection of inflammation *in vivo*. J Nucl Med. (2016) 57:1428–35. 10.2967/jnumed.115.167387 27081166 PMC5093920

[83] Lee WW Marinelli B van der Laan AM Sena BF Gorbatov R Leuschner F. PET/MRI of inflammation in myocardial infarction. J Am Coll Cardiol. (2012) 59:153–63. 10.1016/j.jacc.2011.08.066 22222080 PMC3257823

[84] Lewis AJM Miller JJ Lau AZ Curtis MK Rider OJ Choudhury RP. Noninvasive immunometabolic cardiac inflammation imaging using hyperpolarized magnetic resonance. Circ Res. (2018) 122:1084–93. 10.1161/CIRCRESAHA.117.312535 29440071 PMC5908252

[85] Nunes P Demaurex N Dinauer MC. Regulation of the NADPH oxidase and associated ion fluxes during phagocytosis. Traffic. (2013) 14:1118–31. 10.1111/tra.12115 23980663

[86] Rybicka JM Balce DR Khan MF Krohn RM Yates RM. NADPH oxidase activity controls phagosomal proteolysis in macrophages through modulation of the lumenal redox environment of phagosomes. Proc Natl Acad Sci USA. (2010) 107:10496–501. 10.1073/pnas.0914867107 20498052 PMC2890838

[87] Heckmann BL Boada-Romero E Cunha LD Magne J Green DR. LC3-associated phagocytosis and inflammation. J Mol Biol. (2017) 429:3561–76. 10.1016/j.jmb.2017.08.012 28847720 PMC5743439

[88] Lewis GD Wei R Liu E Yang E Shi X Martinovic M. Metabolite profiling of blood from individuals undergoing planned myocardial infarction reveals early markers of myocardial injury. J Clin Invest. (2008) 118:3503–12. 10.1172/JCI35111 18769631 PMC2525696

[89] Bories GFP Leitinger N. Macrophage metabolism in atherosclerosis. FEBS Lett. (2017) 591:3042–60. 10.1002/1873-3468.12786 28796886

[90] Zhang Z Apse K Pang J Stanton RC. High glucose inhibits glucose-6-phosphate dehydrogenase via cAMP in aortic endothelial cells. J Biol Chem. (2000) 275:40042–7. 10.1074/jbc.M007505200 11007790

[91] Nagareddy PR Murphy AJ Stirzaker RA Hu Y Yu S Miller RG. Hyperglycemia promotes myelopoiesis and impairs the resolution of atherosclerosis. Cell Metab. (2013) 17:695–708. 10.1016/j.cmet.2013.04.001 23663738 PMC3992275

[92] Dutta P Sager HB Stengel KR Naxerova K Courties G Saez B. Myocardial infarction activates CCR2(+) hematopoietic stem and progenitor cells. Cell Stem Cell. (2015) 16:477–87. 10.1016/j.stem.2015.04.008 25957903 PMC4426344

[93] Daffu G Shen X Senatus L Thiagarajan D Abedini A Hurtado Del Pozo C. RAGE suppresses ABCG1-mediated macrophage cholesterol efflux in diabetes. Diabetes. (2015) 64:4046–60. 10.2337/db15-0575 26253613 PMC4657581

[94] Chandel NS. Evolution of mitochondria as signaling organelles. Cell Metab. (2015) 22:204–6. 10.1016/j.cmet.2015.05.013 26073494

[95] Liu X Kim CN Yang J Jemmerson R Wang X. Induction of apoptotic program in cell-free extracts: requirement for dATP and cytochrome c. Cell. (1996) 86:147–57. 10.1016/S0092-8674(00)80085-9 8689682

[96] Chandel NS Maltepe E Goldwasser E Mathieu CE Simon MC Schumacker PT. Mitochondrial reactive oxygen species trigger hypoxia-induced transcription. Proc Natl Acad Sci USA. (1998) 95:11715–20. 10.1073/pnas.95.20.11715 9751731 PMC21706

[97] Murley A Nunnari J. The emerging network of mitochondria-organelle contacts. Mol Cell. (2016) 61:648–53. 10.1016/j.molcel.2016.01.031 26942669 PMC5554544

[98] West AP Brodsky IE Rahner C Woo DK Erdjument-Bromage H Tempst P. TLR signalling augments macrophage bactericidal activity through mitochondrial ROS. Nature. (2011) 472:476–80. 10.1038/nature09973 21525932 PMC3460538

[99] Schagger H Pfeiffer K. Supercomplexes in the respiratory chains of yeast and mammalian mitochondria. EMBO J. (2000) 19:1777–83. 10.1093/emboj/19.8.1777 10775262 PMC302020

[100] Garaude J Acin-Perez R Martinez-Cano S Enamorado M Ugolini M Nistal-Villan E. Mitochondrial respiratory-chain adaptations in macrophages contribute to antibacterial host defense. Nat Immunol. (2016) 17:1037–45. 10.1038/ni.3509 27348412 PMC4994870

[101] Jha AK Huang SC Sergushichev A Lampropoulou V Ivanova Y Loginicheva E. Network integration of parallel metabolic and transcriptional data reveals metabolic modules that regulate macrophage polarization. Immunity. (2015) 42:419–30. 10.1016/j.immuni.2015.02.005 25786174

[102] Lampropoulou V Sergushichev A Bambouskova M Nair S Vincent EE Loginicheva E. Itaconate links inhibition of succinate dehydrogenase with macrophage metabolic remodeling and regulation of inflammation. Cell Metab. (2016) 24:158–66. 10.1016/j.cmet.2016.06.004 27374498 PMC5108454

[103] O'Neill LAJ Artyomov MN. Itaconate: the poster child of metabolic reprogramming in macrophage function. Nat Rev Immunol. (2019). 10.1038/s41577-019-0128-5 30705422

[104] Mills EL Kelly B Logan A Costa ASH Varma M Bryant CE. Succinate dehydrogenase supports metabolic repurposing of mitochondria to drive inflammatory macrophages. Cell. (2016) 167:457–470.e413. 10.1016/j.cell.2016.08.064 27667687 PMC5863951

[105] Zhou R Yazdi AS Menu P Tschopp J. A role for mitochondria in NLRP3 inflammasome activation. Nature. (2011) 469:221–5. 10.1038/nature09663 21124315

[106] Kalogeris T Bao Y Korthuis RJ. Mitochondrial reactive oxygen species: a double edged sword in ischemia/reperfusion vs preconditioning. Redox Biol. (2014) 2:702–14. 10.1016/j.redox.2014.05.006 24944913 PMC4060303

[107] Chouchani ET Pell VR Gaude E Aksentijevic D Sundier SY Robb EL. Ischaemic accumulation of succinate controls reperfusion injury through mitochondrial ROS. Nature. (2014) 515:431–5. 10.1038/nature13909 25383517 PMC4255242

[108] Van den Bossche J Baardman J Otto NA van der Velden S Neele AE van den Berg SM Mitochondrial dysfunction prevents repolarization of inflammatory macrophages. Cell Rep. (2016) 17:684–96. 10.1016/j.celrep.2016.09.008 27732846

[109] Yu E Calvert PA Mercer JR Harrison J Baker L Figg NL. Mitochondrial DNA damage can promote atherosclerosis independently of reactive oxygen species through effects on smooth muscle cells and monocytes and correlates with higher-risk plaques in humans. Circulation. (2013) 128:702–12. 10.1161/CIRCULATIONAHA.113.002271 23841983

[110] Yu EPK Reinhold J Yu H Starks L Uryga AK Foote K. Mitochondrial respiration is reduced in atherosclerosis, promoting necrotic core formation and reducing relative fibrous cap thickness. Arterioscler Thromb Vasc Biol. (2017) 37:2322–32. 10.1161/ATVBAHA.117.310042 28970293 PMC5701734

[111] Foote K Reinhold J Yu EPK Figg NL Finigan A Murphy MP Restoring mitochondrial DNA copy number preserves mitochondrial function and delays vascular aging in mice. Aging Cell. (2018) 2018:e12773 10.1111/acel.12773 PMC6052475 29745022

[112] Ide T Tsutsui H Hayashidani S Kang D Suematsu N Nakamura K. Mitochondrial DNA damage and dysfunction associated with oxidative stress in failing hearts after myocardial infarction. Circ Res. (2001) 88:529–35. 10.1161/01.RES.88.5.529 11249877

[113] Zheng J. Energy metabolism of cancer: Glycolysis versus oxidative phosphorylation (Review). Oncol Lett. (2012) 4:1151–7. 10.3892/ol.2012.928 23226794 PMC3506713

[114] Huang SC Smith AM Everts B Colonna M Pearce EL Schilling JD. Metabolic reprogramming mediated by the mTORC2-IRF4 signaling axis is essential for macrophage alternative activation. Immunity. (2016) 45:817–30. 10.1016/j.immuni.2016.09.016 27760338 PMC5535820

[115] Wang F Zhang S Vuckovic I Jeon R Lerman A Folmes CD Glycolytic stimulation is not a requirement for M2 macrophage differentiation. Cell Metab. (2018) 28:463–75 e464. 10.1016/j.cmet.2018.08.012 PMC6449248 30184486

[116] Huang SC Everts B Ivanova Y O'Sullivan D Nascimento M Smith AM. Cell-intrinsic lysosomal lipolysis is essential for alternative activation of macrophages. Nat Immunol. (2014) 15:846–55. 10.1038/ni.2956 25086775 PMC4139419

[117] Divakaruni AS Hsieh WY Minarrieta L Duong TN Kim KKO Desousa BR. Etomoxir inhibits macrophage polarization by disrupting CoA homeostasis. Cell Metab. (2018) 28:490–503 e497. 10.1016/j.cmet.2018.06.001 30043752 PMC6125190

[118] Nomura M Liu J Rovira II Gonzalez-Hurtado E Lee J Wolfgang MJ. Fatty acid oxidation in macrophage polarization. Nat Immunol. (2016) 17:216–7. 10.1038/ni.3366 26882249 PMC6033271

[119] Bosurgi L Cao YG Cabeza-Cabrerizo M Tucci A Hughes LD Kong Y. Macrophage function in tissue repair and remodeling requires IL-4 or IL-13 with apoptotic cells. Science. (2017) 356:1072–6. 10.1126/science.aai8132 28495875 PMC5556699

[120] Stienstra R Netea-Maier RT Riksen NP Joosten LAB Netea MG. Specific and complex reprogramming of cellular metabolism in myeloid cells during innate immune responses. Cell Metab. (2017) 26:142–56. 10.1016/j.cmet.2017.06.001 28683282

[121] Liu PS Wang H Li X Chao T Teav T Christen S. alpha-ketoglutarate orchestrates macrophage activation through metabolic and epigenetic reprogramming. Nat Immunol. (2017) 18:985–94. 10.1038/ni.3796 28714978

[122] Cull AH Snetsinger B Buckstein R Wells RA Rauh MJ. Tet2 restrains inflammatory gene expression in macrophages. Exp Hematol. (2017) 55:56–70 e13. 10.1016/j.exphem.2017.08.001 28826859

[123] Sano S Oshima K Wang Y MacLauchlan S Katanasaka Y Sano M. Tet2-mediated clonal hematopoiesis accelerates heart failure through a mechanism involving the IL-1beta/NLRP3 inflammasome. J Am Coll Cardiol. (2018) 71:875–86. 10.1016/j.jacc.2017.12.037 29471939 PMC5828038

[124] Fuster JJ MacLauchlan S Zuriaga MA Polackal MN Ostriker AC Chakraborty R. Clonal hematopoiesis associated with TET2 deficiency accelerates atherosclerosis development in mice. Science. (2017) 355:842–7. 10.1126/science.aag1381 28104796 PMC5542057

[125] Jaiswal S Natarajan P Silver AJ Gibson CJ Bick AG Shvartz E. Clonal hematopoiesis and risk of atherosclerotic cardiovascular disease. N Engl J Med. (2017) 377:111–21. 10.1056/NEJMoa1701719 28636844 PMC6717509

[126] Henson PM. Cell removal: efferocytosis. Annu Rev Cell Dev Biol. (2017) 33:127–44. 10.1146/annurev-cellbio-111315-125315 28613937

[127] Howangyin KY Zlatanova I Pinto C Ngkelo A Cochain C Rouanet M. Myeloid-epithelial-reproductive receptor tyrosine kinase and milk fat globule epidermal growth factor 8 coordinately improve remodeling after myocardial infarction via local delivery of vascular endothelial growth factor. Circulation. (2016) 133:826–39. 10.1161/CIRCULATIONAHA.115.020857 26819373 PMC4767109

[128] Wan E Yeap XY Dehn S Terry R Novak M Zhang S. Enhanced efferocytosis of apoptotic cardiomyocytes through myeloid-epithelial-reproductive tyrosine kinase links acute inflammation resolution to cardiac repair after infarction. Circ Res. (2013) 113:1004–12. 10.1161/CIRCRESAHA.113.301198 23836795 PMC3840464

[129] Park D Han CZ Elliott MR Kinchen JM Trampont PC Das S. Continued clearance of apoptotic cells critically depends on the phagocyte Ucp2 protein. Nature. (2011) 477:220–4. 10.1038/nature10340 21857682 PMC3513690

[130] Wang Y Subramanian M Yurdagul AJr Barbosa-Lorenzi VC Cai B de Juan-Sanz J. Mitochondrial Fission Promotes the Continued Clearance of Apoptotic Cells by Macrophages. Cell. (2017) 171:331–45.e22. 10.1016/j.cell.2017.08.041 28942921 PMC5679712

[131] DeBerge M Yeap XY Dehn S Zhang S Grigoryeva L Misener S. MerTK cleavage on resident cardiac macrophages compromises repair after myocardial ischemia reperfusion injury. Circ Res. (2017) 121:930–40. 10.1161/CIRCRESAHA.117.311327 28851810 PMC5623080

[132] Frangogiannis NG. The extracellular matrix in myocardial injury, repair, and remodeling. J Clin Invest. (2017) 127:1600–12. 10.1172/JCI87491 28459429 PMC5409799

[133] Krishnamurthy P Rajasingh J Lambers E Qin G Losordo DW Kishore R. IL-10 inhibits inflammation and attenuates left ventricular remodeling after myocardial infarction via activation of STAT3 and suppression of HuR. Circ Res. (2009) 104:18. 10.1161/CIRCRESAHA.108.188243 19096025 PMC2774810

[134] Ip WKE Hoshi N Shouval DS Snapper S Medzhitov R. Anti-inflammatory effect of IL-10 mediated by metabolic reprogramming of macrophages. Science. (2017) 356:513–9. 10.1126/science.aal3535 28473584 PMC6260791

[135] Zhang S Weinberg S DeBerge M Gainullina A Schipma M Kinchen JM. Efferocytosis fuels requirements of fatty acid oxidation and the electron transport chain to polarize macrophages for tissue repair. Cell Metab. (2019) 29:443–456.e445. 10.1016/j.cmet.2018.12.004 30595481 PMC6471613

[136] Shen Y Kapfhamer D Minnella AM Kim JE Won SJ Chen Y. Bioenergetic state regulates innate inflammatory responses through the transcriptional co-repressor CtBP. Nat Commun. (2017) 8:624. 10.1038/s41467-017-00707-0 28935892 PMC5608947

[137] Schrijvers DM De Meyer GR Kockx MM Herman AG Martinet W. Phagocytosis of apoptotic cells by macrophages is impaired in atherosclerosis. Arterioscler Thromb Vasc Biol. (2005) 25:1256–61. 10.1161/01.ATV.0000166517.18801.a7 15831805

[138] Tabas I. Macrophage death and defective inflammation resolution in atherosclerosis. Nat Rev Immunol. (2010) 10:36–46. 10.1038/nri2675 19960040 PMC2854623

[139] Li S Sun Y Liang CP Thorp EB Han S Jehle AW. Defective phagocytosis of apoptotic cells by macrophages in atherosclerotic lesions of ob/ob mice and reversal by a fish oil diet. Circ Res. (2009) 105:1072–82. 10.1161/CIRCRESAHA.109.199570 19834009 PMC2818555

[140] Yvan-Charvet L Pagler TA Seimon TA Thorp E Welch CL Witztum JL. ABCA1 and ABCG1 protect against oxidative stress-induced macrophage apoptosis during efferocytosis. Circ Res. (2010) 106:1861–9. 10.1161/CIRCRESAHA.110.217281 20431058 PMC2995809

[141] Viaud M Ivanov S Vujic N Duta-Mare M Aira LE Barouillet T. Lysosomal cholesterol hydrolysis couples efferocytosis to anti-inflammatory oxysterol production. Circ Res. (2018) 122:1369–84. 10.1161/CIRCRESAHA.117.312333 29523554 PMC6034181

[142] Wolfson RL Sabatini DM. The dawn of the age of amino acid sensors for the mTORC1 pathway. Cell Metab. (2017) 26:301–9. 10.1016/j.cmet.2017.07.001 28768171 PMC5560103

[143] Zhenyukh O Civantos E Ruiz-Ortega M Sanchez MS Vazquez C Peiro C. High concentration of branched-chain amino acids promotes oxidative stress, inflammation and migration of human peripheral blood mononuclear cells via mTORC1 activation. Free Radic Biol Med. (2017) 104:165–77. 10.1016/j.freeradbiomed.2017.01.009 28089725

[144] Ikeda K Kinoshita M Kayama H Nagamori S Kongpracha P Umemoto E. Slc3a2 mediates branched-chain amino-acid-dependent maintenance of regulatory T Cells. Cell Rep. (2017) 21:1824–38. 10.1016/j.celrep.2017.10.082 29141216

[145] Raffel S Falcone M Kneisel N Hansson J Wang W Lutz C. BCAT1 restricts alphaKG levels in AML stem cells leading to IDHmut-like DNA hypermethylation. Nature. (2017) 551:384–8. 10.1038/nature24294 29144447

[146] Papathanassiu AE Ko JH Imprialou M Bagnati M Srivastava PK Vu HA. BCAT1 controls metabolic reprogramming in activated human macrophages and is associated with inflammatory diseases. Nat Commun. (2017) 8:16040. 10.1038/ncomms16040 28699638 PMC5510229

[147] Kwon NS Nathan CF Gilker C Griffith OW Matthews DE Stuehr DJ. L-citrulline production from L-arginine by macrophage nitric oxide synthase. The ureido oxygen derives from dioxygen. J Biol Chem. (1990) 265:13442–5. 1696255

[148] Granger DL Hibbs JB Jr Perfect JR Durack DT. Specific amino acid (L-arginine) requirement for the microbiostatic activity of murine macrophages. J Clin Invest. (1988) 81:1129–36. 10.1172/JCI113427 3280600 PMC329641

[149] Hibbs JB Jr Taintor RR Vavrin Z. Macrophage cytotoxicity: role for L-arginine deiminase and imino nitrogen oxidation to nitrite. Science. (1987) 235:473–6. 10.1126/science.2432665 2432665

[150] Qualls JE Subramanian C Rafi W Smith AM Balouzian L DeFreitas AA. Sustained generation of nitric oxide and control of mycobacterial infection requires argininosuccinate synthase 1. Cell Host Microbe. (2012) 12:313–23. 10.1016/j.chom.2012.07.012 22980328 PMC3444824

[151] El Kasmi KC Qualls JE Pesce JT Smith AM Thompson RW Henao-Tamayo M Toll-like receptor-induced arginase 1 in macrophages thwarts effective immunity against intracellular pathogens. Nat Immunol. (2008) 9:1399–406. 10.1038/ni.1671 18978793 PMC2584974

[152] Schulman SP Becker LC Kass DA Champion HC Terrin ML Forman S. L-arginine therapy in acute myocardial infarction: the Vascular Interaction With Age in Myocardial Infarction (VINTAGE MI) randomized clinical trial. JAMA. (2006) 295:58–64. 10.1001/jama.295.1.58 16391217

[153] Kirk SJ Hurson M Regan MC Holt DR Wasserkrug HL Barbul A. Arginine stimulates wound healing and immune function in elderly human beings. Surgery. (1993) 114:155–9; discussion 160. 8342121

[154] Campbell L Saville CR Murray PJ Cruickshank SM Hardman MJ. Local arginase 1 activity is required for cutaneous wound healing. J Invest Dermatol. (2013) 133:2461–70. 10.1038/jid.2013.164 23552798 PMC3778883

[155] Gerriets VA Kishton RJ Nichols AG Macintyre AN Inoue M Ilkayeva O. Metabolic programming and PDHK1 control CD4+ T cell subsets and inflammation. J Clin Invest. (2015) 125:194–207. 10.1172/JCI76012 25437876 PMC4382238

[156] O'Neill LA Kishton RJ Rathmell J. A guide to immunometabolism for immunologists. Nat Rev Immunol. (2016) 16:553–65. 10.1038/nri.2016.70 27396447 PMC5001910

[157] Arts RJ Novakovic B Ter Horst R Carvalho A Bekkering S Lachmandas E. Glutaminolysis and fumarate accumulation integrate immunometabolic and epigenetic programs in trained immunity. Cell Metab. (2016) 24:807–19. 10.1016/j.cmet.2016.10.008 27866838 PMC5742541

[158] He L Weber KJ Schilling JD. Glutamine modulates macrophage lipotoxicity. Nutrients. (2016) 8:215. 10.3390/nu8040215 27077881 PMC4848684

[159] Khogali SE Pringle SD Weryk BV Rennie MJ. Is glutamine beneficial in ischemic heart disease? Nutrition. (2002) 18:123–6. 10.1016/S0899-9007(01)00768-7 11844641

[160] Hotamisligil GS. Endoplasmic reticulum stress and the inflammatory basis of metabolic disease. Cell. (2010) 140:900–17. 10.1016/j.cell.2010.02.034 20303879 PMC2887297

[161] Hardie DG Ross FA Hawley SA. AMPK: a nutrient and energy sensor that maintains energy homeostasis. Nat Rev Mol Cell Biol. (2012) 13:251–62. 10.1038/nrm3311 22436748 PMC5726489

[162] Yeung F Hoberg JE Ramsey CS Keller MD Jones DR Frye RA. Modulation of NF-kappaB-dependent transcription and cell survival by the SIRT1 deacetylase. Embo J. (2004) 23:2369–80. 10.1038/sj.emboj.7600244 15152190 PMC423286

[163] Kimura N Tokunaga C Dalal S Richardson C Yoshino K Hara K. A possible linkage between AMP-activated protein kinase (AMPK) and mammalian target of rapamycin (mTOR) signalling pathway. Genes Cells. (2003) 8:65–79. 10.1046/j.1365-2443.2003.00615.x 12558800

[164] Filippone SM Samidurai A Roh SK Cain CK He J Salloum FN. Reperfusion therapy with rapamycin attenuates myocardial infarction through activation of AKT and ERK. Oxid Med Cell Longev. (2017) 2017:4619720. 10.1155/2017/4619720 28373901 PMC5360974

[165] Zinman B Wanner C Lachin JM Fitchett D Bluhmki E Hantel S. Empagliflozin, cardiovascular outcomes, and mortality in type 2 diabetes. N Engl J Med. (2015) 373:2117–28. 10.1056/NEJMoa1504720 26378978

[166] Palsson-McDermott EM Curtis AM Goel G Lauterbach MAR Sheedy FJ Gleeson LE Pyruvate kinase M2 regulates Hif-1alpha activity and IL-1beta induction and is a critical determinant of the warburg effect in LPS-activated macrophages. Cell Metab. (2015) 21:347 10.1016/j.cmet.2015.01.017 29510100

[167] Dror E Dalmas E Meier DT Wueest S Thevenet J Thienel C. Postprandial macrophage-derived IL-1beta stimulates insulin, and both synergistically promote glucose disposal and inflammation. Nat Immunol. (2017) 18:283–92. 10.1038/ni.3659 28092375

[168] Frangogiannis NG. Interleukin-1 in cardiac injury, repair, and remodeling: pathophysiologic and translational concepts. Discoveries. (2015) 3:33. 10.15190/d.2015.33 26273700 PMC4532433

[169] Guillen I Blanes M Gomez-Lechon MJ Castell JV. Cytokine signaling during myocardial infarction: sequential appearance of IL-1 beta and IL-6. Am J Physiol. (1995) 269:R229–235. 10.1152/ajpregu.1995.269.2.R229 7544543

[170] Sager HB Heidt T Hulsmans M Dutta P Courties G Sebas M. Targeting interleukin-1beta reduces leukocyte production after acute myocardial infarction. Circulation. (2015) 132:1880–90. 10.1161/CIRCULATIONAHA.115.016160 26358260 PMC4651795

[171] Ridker PM Everett BM Thuren T MacFadyen JG Chang WH Ballantyne C. Antiinflammatory therapy with canakinumab for atherosclerotic disease. N Engl J Med. (2017) 377:1119–31. 10.1056/NEJMoa1707914 28845751

[172] Ridker PM MacFadyen JG Thuren T Everett BM Libby P Glynn RJ. Effect of interleukin-1beta inhibition with canakinumab on incident lung cancer in patients with atherosclerosis: exploratory results from a randomised, double-blind, placebo-controlled trial. Lancet. (2017) 390:1833–42. 10.1016/S0140-6736(17)32247-X 28855077

[173] Gomez D Baylis RA Durgin BG Newman AAC Alencar GF Mahan S. Interleukin-1beta has atheroprotective effects in advanced atherosclerotic lesions of mice. Nat Med. (2018) 24:1418–29. 10.1038/s41591-018-0124-5 30038218 PMC6130822

[174] Koelwyn GJ Corr EM Erbay E Moore KJ. Regulation of macrophage immunometabolism in atherosclerosis. Nat Immunol. (2018) 19:526–37. 10.1038/s41590-018-0113-3 29777212 PMC6314674

[175] Ridker PM Everett BM Pradhan A MacFadyen JG Solomon DH Zaharris E. Low-dose methotrexate for the prevention of atherosclerotic events. N Engl J Med. (2018) 380:752–62. 10.1056/NEJMoa1809798 30415610 PMC6587584

[176] Guilliams M Mildner A Yona S. Developmental and Functional Heterogeneity of Monocytes. Immunity. (2018) 49:595–613. 10.1016/j.immuni.2018.10.005 30332628

[177] Zhang H Xue C Shah R Bermingham K Hinkle CC Li W. Functional analysis and transcriptomic profiling of iPSC-derived macrophages and their application in modeling Mendelian disease. Circ Res. (2015) 117:17–28. 10.1161/CIRCRESAHA.117.305860 25904599 PMC4565503

[178] Rymer JA Newby LK. Failure to launch: targeting inflammation in acute coronary syndromes. JACC Basic Trans Sci. (2017) 2:484–97. 10.1016/j.jacbts.2017.07.001 30062164 PMC6034453

